# Responding to Other People’s Direct Gaze: Alterations in Gaze Behavior in Infants at Risk for Autism Occur on Very Short Timescales

**DOI:** 10.1007/s10803-017-3253-7

**Published:** 2017-09-04

**Authors:** Pär Nyström, Sven Bölte, Terje Falck-Ytter, The EASE Team, Sheila Achermann, Linn Andersson Konke, Karin Brocki, Elodie Cauvet, Gustaf Gredebäck, Johan Lundin Kleberg, Elisabeth Nilsson Jobs, Emilia Thorup, Eric Zander

**Affiliations:** 1 Uppsala Child & Babylab, Department of Psychology, Uppsala University, Box 1225, 75142 Uppsala, Sweden; 2 Center of Neurodevelopmental Disorders (KIND), Division of Neuropsychiatry, Department of Women’s & Children’s Health, Karolinska Institutet, Stockholm, Sweden; 3 Child and Adolescent Psychiatry, Center for Psychiatry Research, Stockholm County Council, Stockholm, Sweden

**Keywords:** Sensorimotor development, Neurodevelopmental disorders, Risk assessment, Eye tracking, Autism

## Abstract

Atypical gaze processing has been reported in children with autism spectrum disorders (ASD). Here we explored how infants at risk for ASD respond behaviorally to others’ direct gaze. We assessed 10-month-olds with a sibling with ASD (high risk group; n = 61) and a control group (n = 18) during interaction with an adult. Eye-tracking revealed less looking at the adult in the high risk group during 300–1000 ms after the adult initiated direct gaze: a short alteration that is likely to go unnoticed by the naked eye. Data aggregated over longer segments (the traditional eye-tracking approach) showed no group differences. Although findings are limited by lack of outcome data, they are in line with theories linking atypical eye processing to the emergence of ASD.

**Electronic supplementary material:**

The online version of this article (doi:10.1007/s10803-017-3253-7) contains supplementary material, which is available to authorized users.

## Introduction

Altered social interaction and communication are core features of autism spectrum disorders (ASD) and already has Leo Kanner noted unusual eye contact and markedly reduced looking at others’ eyes in his seminal report (Kanner 1943). Direct gaze processing, which is a basic and fundamental part of social interplay (Csibra and Gergely 2006; Kleinke 1986; Senju et al. 2008), has received much attention in ASD research, exemplified by Dalton et al. (2005), Senju and Johnson (2009a), and many others. This and other research suggests that alterations in direct gaze processing could preceed behaviors often reported as atypical in ASD, such as gaze avoidance, altered gaze following, impaired joint attention, and even theory of mind (von dem Hagen et al. 2013; Senju and Johnson 2009b). It is therefore important to study response to direct gaze as early in development as possible.

Response to other people’s direct gaze develops during early infancy but ASD is rarely diagnosed before 2 years of age. Therefore prospective studies are indispensable to deciphering the potential association between response to direct gaze early in life and subsequent ASD diagnosis. As the recurrence risk in siblings of children with ASD may be as high as ~20% (as compared to ~1% in the general population; Messinger et al. 2015), longitudinal sibling research provides an efficient strategy to study early signs of ASD (Jones et al. 2014). For example, Elsabbagh et al. (2009) showed that neural processing has prolonged latencies during passive viewing of static computer generated faces with direct gaze in ~10 month old infants at risk for ASD (i.e. the younger siblings) compared to typically developing infants.

In a related study, Elsabbagh et al. (2012) assessed infants at risk (6–10 months of age) using both static and dynamic computer stimuli of a face alternating between direct and averted gaze. The results showed significant differences in neural activation between the high risk group and a control group for both static stimuli (latency differences) and dynamic stimuli (both latency and amplitude differences) in response to direct gaze. The researchers then followed the infants until 3 years of age, and categorized the infants into four groups: control, At-risk no ASD, At-risk ASD, and At-risk early ASD. The results for these groups showed that only the two non-autistic outcome groups (control and At-risk no ASD) differentiated between dynamic direct and averted gaze in the neural response, and that the differences were most pronounced ~400 ms after direct gaze. Thus, reduced neural sensitivity to dynamic eye gaze in infancy is associated with later emerging ASD. Importantly, when static pictures of direct and averted gaze were compared in the outcome groups, or when static pictures of faces were compared to noise, the ASD groups could not be reliably distinguished from the two non-autistic groups according to the authors. However, there was a within-group latency difference between faces and noise in the At-risk ASD group that was absent in the other groups (a finding that has been replicated in another study), again suggesting that fine grained temporal analysis is important. Also, a separate eye tracking control experiment did not show any differences in overall looking times at the eye region between groups. Together this suggests that altered processing of direct gaze is more likely to manifest in response to dynamic and thus more ecologically valid situations than in static presentations, and that analyzing behavior on short timescales could be required to detect subtle behavioral markers in this context.

The EEG studies above did not assess the behavior of the infants in response to direct gaze, such as establishing or avoiding eye contact. An important remaining question is thus whether or not altered *behavioral* responses to direct gaze are present in infants at risk for ASD. Behavioral alterations that are present early and which could affect the pattern of interaction with others in tangible ways are particularly important to document, as they are most likely to lead to cascading developmental effects in interaction with the environment (Krstovska-Guerrero and Jones 2016). For example, if an infant does not look back when the parent tries to initiate eye contact, the parent may feel less rewarded and engage less in direct gaze un the future. Then, the infant’s input may be impoverished too, leading to a deteriorating loop (Dawson et al. 2004).

Several studies are available showing that individual differences in gaze patterns in infancy relate to later development in ASD as well as in typical developing children. For example, one study found that looking time at the mother’s mouth in infancy was associated with larger vocabulary in toddlerhood, suggesting that early attention to rich and continous (visual and audiovisual) information available from this part of the face may have boosted the development of language skills (Young et al. 2009), and for related face processing studies focusing on the still face procedure, see Merin et al. 2007; Cassel et al. 2007. Studies of joint attention have also shown a link between gaze behavior and language aquisision (Morales et al. 2000; Yoder et al. 2006), and documented early alterations in infants at risk for autism (Presmanes et al. 2007; Thorup et al. 2016). While these studies [and many others, including Mundy and Newell (2007); Mundy and Crowson (1997); Bedford et al. (2012), etc.] indicate early atypicalities in socio-communicative skills in ASD, they have not assessed how infants at risk respond to other people’s direct gaze, i.e. a very basic visual signal of communicative intent.

Another limitation of previous research is the lack of naturalistic study settings, which renders the ecological validity of the studies unclear (Foulsham et al. 2011; Risko et al. 2012; Freeth et al. 2013; Laidlaw et al. 2011; Redcay et al. 2010; Falck-Ytter 2015; Falck-Ytter et al. 2015). Live presentation of human behavior elicits stronger neural responses than televised presentation (Redcay et al. 2010; Shimada and Hiraki 2006; Järveläinen et al. 2001; Jones et al. 2015), suggesting that behavioral responses are more likely to arise from social stimuli presented live than on a monitor. Although there are retrospective reports using home videos showing atypical eye contact in ASD during the first year of life (Maestro et al. 2002, 2005; Mars et al. 1998), those studies are hampered by general concerns for retrospective designs, such as unsystematic data collection or no experimental manipulation, and hence difficulties to deduce strong conclusions. Additionally, a study by Ozonoff et al. (2010) used video coded material to demonstrate emerging differences in overall “Gaze to Faces” between 6 and 12 months of age in children later diagnosed with ASD, but did not include any specific references to direct gaze. To the best of our knowledge, no published study has investigated the behavioral response to direct gaze using specifically designed tasks in natural settings.

In the current study we investigated whether infants at risk for ASD show altered behavioral response to another person’s direct gaze during face-to-face social interaction. We hypothesized, based on previous EEG studies (Elsabbagh et al. 2012, 2009), that infants at risk for ASD would spend less time looking at another person shortly after onset of direct gaze initiation compared to typically developing infants. The administered task is part of Early Autism Sweden (EASE), an ongoing longitudinal study with infants having older siblings with ASD. The data reported in this study were collected during the EASE 10 month assessment.

## Methods

### Participants

Infants in the high risk for ASD group (n = 65, 35 girls) were recruited from the EASE project website, advertisements in the journals of interest organizations, and via clinical compulsory care units. Infants in the low risk for ASD group (n = 24, 9 girls) were recruited from state birth records. All infants were born full-term (>36 weeks) and did not have any confirmed or suspected medical conditions, including visual/auditory impairments. Both groups were living primarily in the greater urban areas of Stockholm, Sweden. Parents provided written informed consent and the study was approved by the Regional Ethical Board in Stockholm. The study was conducted in accordance with the standards specified in the 1964 Declaration of Helsinki. All families received a compensation of €50 for their participation in the study.

The two groups did not differ in terms of chronological and cognitive/motor developmental age as well as socioeconomic background (Table 1). We used The Mullen Scales of Early Learning (MSEL) (Mullen 1995) to assess developmental level. Socioeconomic background was determined based on family income and parental education level. In the high risk ASD group, we confirmed the ASD diagnosis of the older sibling by reviewing medical records, where at least 70% of all diagnoses were based on the Autism Diagnostic Observation Schedule (ADOS) (Lord et al. 2012) and/or the Autism Diagnostic Interview-Revised (ADI-R) (Le Couteur et al. 2003). In the remaining cases, information regarding the use of these instruments was not present. Low risk ASD infants had no relatives (up to second degree) with ASD. To match the high risk ASD group with the low risk group we also required that all low risk infants had at least one typically developing older sibling.

**Table 1:** Participant characteristics by ASD risk group, final samples (mean/SD)

Measure	High risk ASD group N = 60	Low risk ASD group N = 18	Pairwise comparison (*p* value^a^)
Age (months)	10.41/0.46	10.41/0.54	0.960
MSEL^b^ total score	100.77/13.47	100.333/12.00	0.902
MSEL VR^c^	54.69/10.15	55.83/6.19	0.652
MSEL FM^d^	56.02/9.10	57.00/11.09	0.703
MSEL RL^e^	45.54/9.52	43.06/11.40	0.355
MSEL EL^f^	45.15/9.97	42.11/11.22	0.273
SES^g^	7.07/2.01	7.88/1.65	0.132

^a^Independent samples *t* test

^b^Mullen scales of early learning

^c^Visual reception subscale

^d^Fine motor subscale

^e^Receptive language subscale

^f^Expressive language subscale

^g^Socio-economic status calculated on the basis of parental education and income (Approximate income bands: <1000 EUR; 1000–2000 EUR; 2000–3000 EUR; 3000–4000 EUR; 4000–5000 EUR; >5000 EUR. Education bands: elementary school; 2 years secondary education; 3–4 years secondary education; <3 years higher education; ≥3 years higher education; for this measure, N = 58 in the high risk ASD group and N = 17 in the low risk ASD group because some families did not disclose this information)

### Procedure and stimuli

After the parents arrived to the lab, a test leader explained the purpose of the general study and described the various elements of the visit. Participation in the study typically involved 4–5 h of assessments from a large set of experimental protocols, interleaved with pauses, naps, and lunch. The test leader and a clinical psychologist typically guided the participants for the whole visit, making the full blinding of group status difficult. However, the test leader and the psychologist were instructed not to talk about the familial history of ASD with the parent, unless this was brought up by the parent. To reduce the risk that this occurred and to maximize attention to the task, the eye tracking task reported here was administered as early as possible (usually around 10:30 AM, after ~20 min of information and accommodation to the lab), with the MSEL following directly after.

In the eye tracking session, the infant was seated in the parent’s lap at a distance of ~200 cm from a test leader sitting behind a low table (the same setup as Thorup et al. 2016, see Supplementary Information for an illustration). The gaze of the infant was recorded using a Tobii TX300 eye tracker without a monitor, which is suitable for infant eye tracking with live stimuli, placed between the infant and the test leader. The eye tracker sampled at 120 Hz. A scene camera recorded the test leader during the whole session with a sampling rate of 30 frames per second and a resolution of 640 × 360 pixels. The scene camera recordings were later used for offline behavior coding. Before starting the eye tracking recording the test leader performed a standard 5-point calibration by moving a squeaky toy across predefined calibration points in the same plane as where the test leader would later be seated. After calibration the test leader performed a battery of tasks for a duration of approximately 8–10 min as in Thorup et al. 2016. Only the task designed to assess the response to direct gaze is presented in the current paper.

**Fig. 1:**
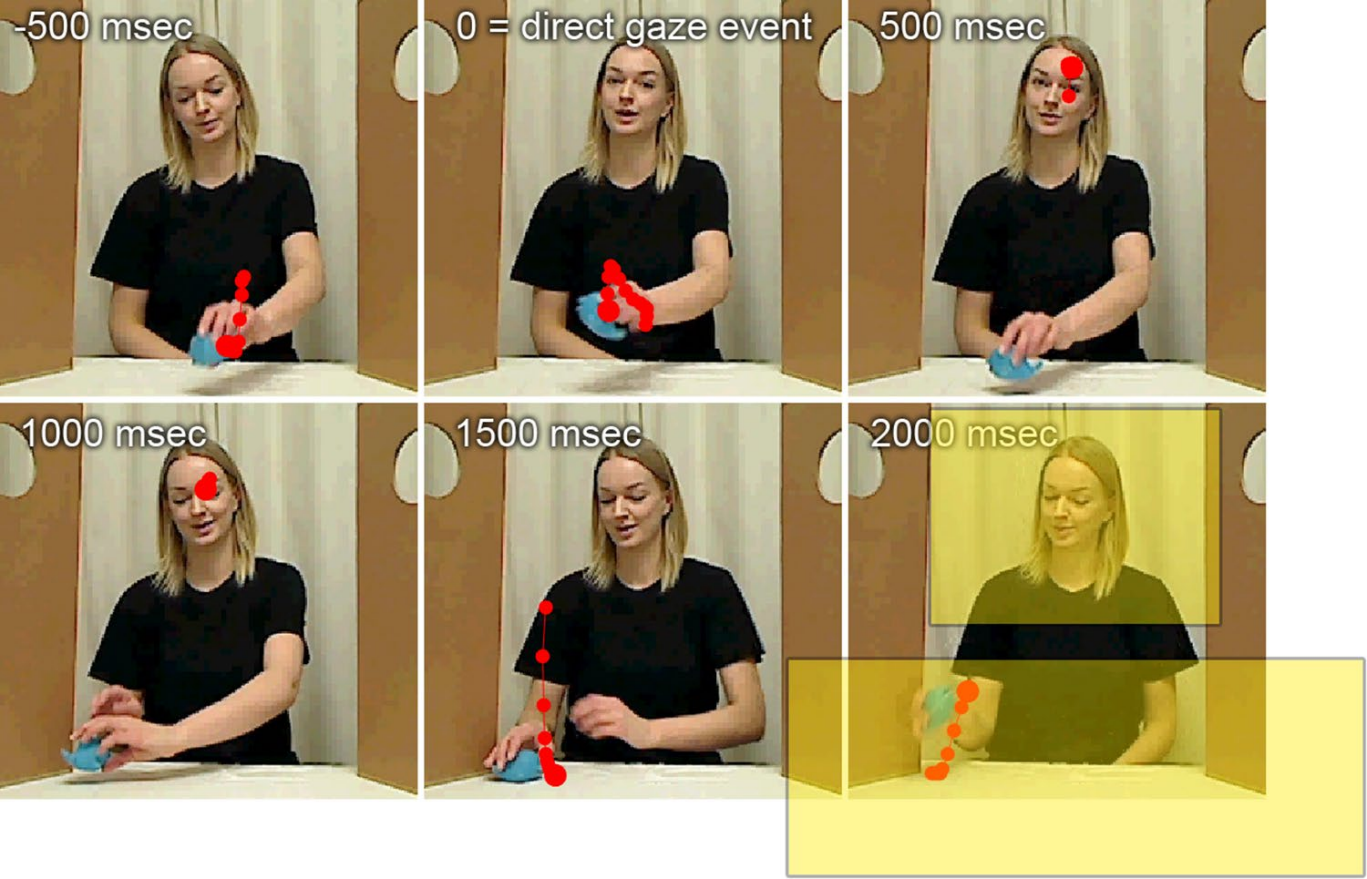
Example of test leader performing the direct gaze response task. *Red dots mark* an observing infant’s gaze samples around the timestamp. Uncropped AOIs are superimposed on the bottom right cropped video frame. (Color figure online)

We created a situation where the role of direct gaze could be studied during a naturalistic social interaction. The procedure was as follows: The test leader introduced an object (a toy car or a toy fish) to the infant and started to play with the object by moving it across the table and making playful sounds, such as imitating the motor sound of a car or saying ‘blob-blob’, intended to be the sound of the fish. The test leader looked primarily at the object, interrupted by several shorter looks towards the infant referred to as the “direct gaze event”, in order to show that he or she was aware of the infant and intended to share his/her experience with the infant in a natural way. These direct events were later used when extracting trials (see below for procedure and descriptive statistics).The test leader generally still made the same playful sound during the direct gaze event as when s/he looked at the toy. The test leader performed at least two direct gaze events before making the car crash into a small wall, an event which is followed by a direct gaze event and a short monologue to keep the infants’ attention (similar to ‘Oops! I wonder if everything is OK with the car/fish? I hope so… OK, let’s continue playing!’). The test leader continued to play with the object and repeated the crash once more, before ending the play by removing the object from the table. A video example of the procedure can be found in the Supplementary Materials. The direct gaze response task took approximately 30–40 s, and was performed twice for each infant (the first time with the car and the second time with the fish), separated by a calibration quality assessment and other tasks (Thorup et al. 2016) within the same eye tracking session. An example of the direct gaze task is found in Fig. 1. In the current dataset the first direct gaze block started on average 1.5 min after the recording started (high ASD risk group: M = 86.0 s, SD = 16.2; low risk group: M = 90.4 s, SD = 15.2), and the second block started on average 5.7 min after the start of the recording (high ASD risk group: M = 333.6 s, SD = 58.6; low risk group: M = 364.1 s, SD = 76.6). The total duration of the two direct gaze blocks averaged 86.2 s (SD = 19.7) in the high ASD risk group and 96.3 s (SD = 21.1) in the low risk group.

Six different test leaders (2 male, 4 female) performed the direct gaze task in order to increase social variability and thus generalizability. One male test leader tested only one infant, and this infant was removed from analysis. The female test leaders tested 47 high ASD risk infants and 11 low risk infants. The remaining male test leader tested 13 high ASD risk infants and 7 low risk infants. The task administration procedure was standardized to minimize influences of individual interaction styles, and each test leader was trained extensively according to a video template of the whole session before performing actual recordings (relevant video excerpt is found in the Supplementary Material). Test leader identity was stored as a nominal variable and later used in the statistical analysis.

### Control Eye Tracking Task: Visual Disengagement

The test leaders also administered a disengagement task between the two direct gaze response blocks, similar to the one used in the Autism Observation Scale for Infants (AOSI). The disengagement task included two control measures of visual disengagement which were later used in the statistical analyses: (1) the disengagement latency from one object to another, and (2) the variability of disengagement latencies. The procedure of the disengagement task and the two control measures are described in more detail in the Supplementary Information.

### Data Analysis

The gaze behavior of the test leader was coded offline using the Tobii Studio Software and the scene camera recording on a frame-by-frame basis. The gaze behavior of the test leader was categorized by change in looking direction (left/right/down/direct gaze), and these events were integrated together into the gaze data files with timestamps. Data files were exported to text format and imported into MATLAB R2015a and analyzed using the TimeStudio framework (Nyström et al. 2016). The complete analysis workflow can be downloaded from TimeStudio using uwid ts-ad5-b91. Due to privacy reasons the data files and assessment data have been removed from this workflow.

Three areas of interest (AOIs) were defined that covered the table and the object (toy AOI, 400 × 150 pixels), the test leader’s face (face AOI, 200 × 150 pixels), and the entire scene (scene AOI, 640 × 360 pixels). Similar to previous studies using this set up (i.e. child seated at a distance of 200 cm from the test leader Thorup et al. 2016, we refrained from specifying separate eye and mouth AOIs, due to the risk that these would not generate reliable data from a sufficient number of children—instead, we choose a more conservative approach defining a single large AOI that covered the whole face and head.

Trials were defined as starting 500 ms before each time a test leader initiated a direct look at the infant (direct gaze events) and stopping 2000 ms after the event. Some trials contained missing gaze data due to the infants sometimes moving or looking away during the recording session. Trials with more than 50% missing data were considered unreliable and excluded from the analysis. Missing gaze data were linearly interpolated in the other trials. On average, in the high risk ASD group, 2.6 trials were rejected per participant (SD = 3.6) and in the low risk ASD group 3.6 trials (SD = 3.7), which was not a significant difference according to an independent *t* test (p = .34). Infants with less than two trials after trial rejection were excluded from further analyses. All excluded infants were determined to have low data quality from the eye tracker. The final sample (Table 1) consisted of 60 high ASD risk infants with 8–29 trials (M = 18.2, SD = 4.7) and 18 low risk infants with 12–29 trials (M = 21.1, SD = 4.7). The number of valid trials was stored as an interval variable for later analysis. The time between trials was calculated, giving an average time of 5.1 s (SD = 1.8) in the high ASD risk group and 4.4 s (SD = 0.7) in the low risk group.

In each trial, the percent looking time within the face AOI (calculated as the number of samples in face AOI/number of samples in scene AOI) was calculated for 100 ms time bins (26 time bins labeled tb1–tb26), effectively creating a time series signal of preference to the test leader face.

A baseline was calculated for each trial, which was defined as the average of the first five time bins (i.e., the 500 ms before the direct gaze event). We calculated the main dependent measure in the study, “change in face preference” (ΔFacePref), by subtracting the baseline from the face preference data in all subsequent time bins. Thus, if the infants respond by meeting the adult’s direct gaze and start looking at the face AOI, then ΔFacePref will increase shortly after the direct gaze event (time = 0 ms). The baseline correction was performed to reduce inter-infant variability while allowing the inclusion of all trials (both when the infant looked at the test leader at trial start, and when the infant looked at the toy). The baseline value was stored and used a controlling variable in the statistical analysis.

We also calculated two other measures that circumvented the baseline correction by only using trials with a baseline >80% (i.e. when the infant was looking at the test leader at the onset of direct gaze) and trials with a baseline <20% (i.e. the infant was looking at the toy and seeing the test leader in the periphery). The statistical analyses using these measures have not included as many infants and trials, and thus less power, but depict the gaze behavior in two different situations: one in which gaze processing occurs mainly in the central visual field, and one in which it occurs mainly in the peripheral visual field respectively.

### Statistical Analysis

Based on typical latencies of reactive saccades (Gredebäck et al. 2006), we expected ΔFacePref to increase no earlier than ~100 ms. Although we suspected that the effect could be short lived (Elsabbagh et al. 2012; Falck-Ytter et al. 2013) we did not have any exact expectations for the duration of the putative response. To determine time windows with differences between high risk and low risk ASD groups we applied two-tailed independent *t* tests for each time bin, and corrected for multiple testing by controlling the false discovery rate (FDR, Benjamini–Hochberg procedure) with an alpha threshold of 0.1. This alpha threshold was justified by the directionality of the study hypothesis (it gives equivalent results to one-tailed *t* tests and a FDR threshold of 0.05).

The time window with significant differences were further analyzed with a stepwise linear regression model (with a bidirectional elimination procedure) to understand the potential role of various covariates. First, ΔFacePref as a response variable and group as predictor variable were added to the model. Then, possibly confounding variables and their interaction terms were added if the F-test for the confounding predictor had a p value of 0.1 or less. Additional predictors were (1) baseline value, (2) AOSI score, (3) Mullen score, (4) disengagement latency, (5) disengagement standard deviation, (6) the AOSI item “Orients to name”, (7) number of valid trials, (8) number of trials per second, and (9) test leader identity; see supplementary information for more information about definitions of these predictors. If no terms could be added, then the terms currently in the model were examined, and the worst one was removed if an F-test for removing it had a p value .2 or greater. The process was repeated until no terms could be added or removed. This selection procedure may inflate type I errors, but because the added predictors were control variables we opted for the conservative strategy of rather including too many control variables than too few. Despite this, only test leader identity without the interaction term was included in the final model: “*ΔFacePref* ~ *group* + *test leader identity”*.

All inferential statistical analyses were performed in MATLAB using the Statistical Toolbox, and F-test statistics using hierarchical type III sum of squares are reported. All residuals were normally distributed according to Kolmogorov–Smirnov tests and residual plots, and there were no autocorrelation according to Durbin–Watson test.

## Results

### Defining the Time Interval of Interest

**Fig. 2:**
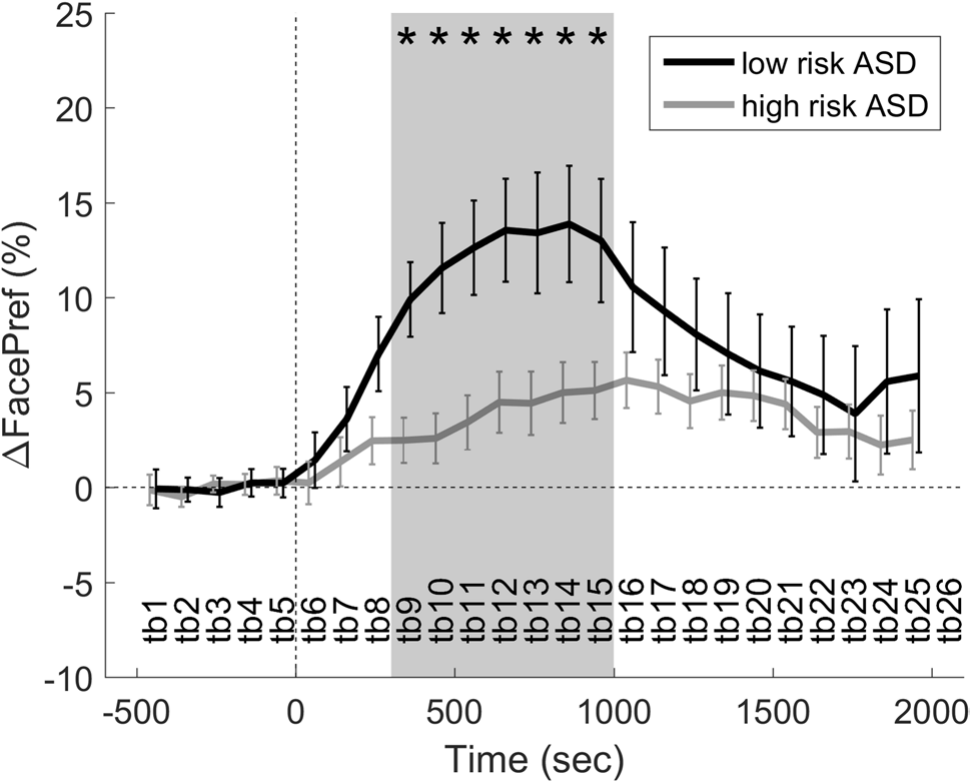
Change in face preference (ΔFacePref) following direct gaze events (time = 0 s). *Error bars* are ±SEM. *Grey* interval indicates the time bins with significant differences between groups that was subsequently averaged and tested in a linear regression model. *Asterisks* individual time bins with a significant group difference

Independent *t* test were used to find time bins with differences in face preference between groups. Significant differences corrected for multiple testing were found between 300 and 1000 ms after direct gaze, with significantly more looking time at the test leader face in the low risk ASD group compared to the high risk ASD group (see Fig. 2).

### Main Analyses

The identified interval 300–1000 ms was averaged within infants as their ΔFacePref in response to direct gaze from the test leader. A linear regression model specified as “ΔFacePref ~ group + test leader identity” (see “Methods”) was fitted which showed a significant main effect of group, F(1, 72) = 9.78, p = .003, R^2^ = .101. Descriptive statistics for ΔFacePref split by group was: high ASD risk n = 60, M = 3.93, SEM = 1.31, 95% CI 1.31–6.55; low ASD risk n = 18, M = 12.56, SEM = 2.48, 95% CI 7.34–17.79. There was also a main effect of model identity, F(4, 72) = 3.83, p = .007, R^2^ = .158.

### Stability of Effect Across Different Test Leaders

The main effect for test leader ID was unexpected and descriptive statistics for ΔFacePref split by test leader identify was: female1 n = 33, M = 7.19, SEM = 1.83, 95% CI 3.46–10.91; female2 n = 10, M = −5.29, SEM = 2.34, 95% CI −10.58–0.01; female3 n = 7, M = 7.77, SEM = 4.02–11.51; female4 n = 8, M = 5.21, SEM = 3.84, 95% CI −3.87–14.30; male1 n = 20, M = 9.07, SEM = 2.57, 95% CI 3.69–14.44. The descriptive statistics showed that test leader female2 had lower ΔFacePref, and another linear regression was performed where female2 was removed from the analysis. This time the linear regression analysis only showed a significant main effect of groups: F(1, 63) = 9.069, p = .004, R^2^ = .125, and model identify was no longer significant: F(3, 63) = 0.066, p = .978, R^2^ = .003. Thus, because the main group difference remains even when excluding the test leader who elicited different responses, and because there was no significant interaction between group and model identity, the difference between groups appears to be robust to different test leader interaction styles.

### Direct Gaze in Central Versus Peripheral Vision

**Fig. 3:**
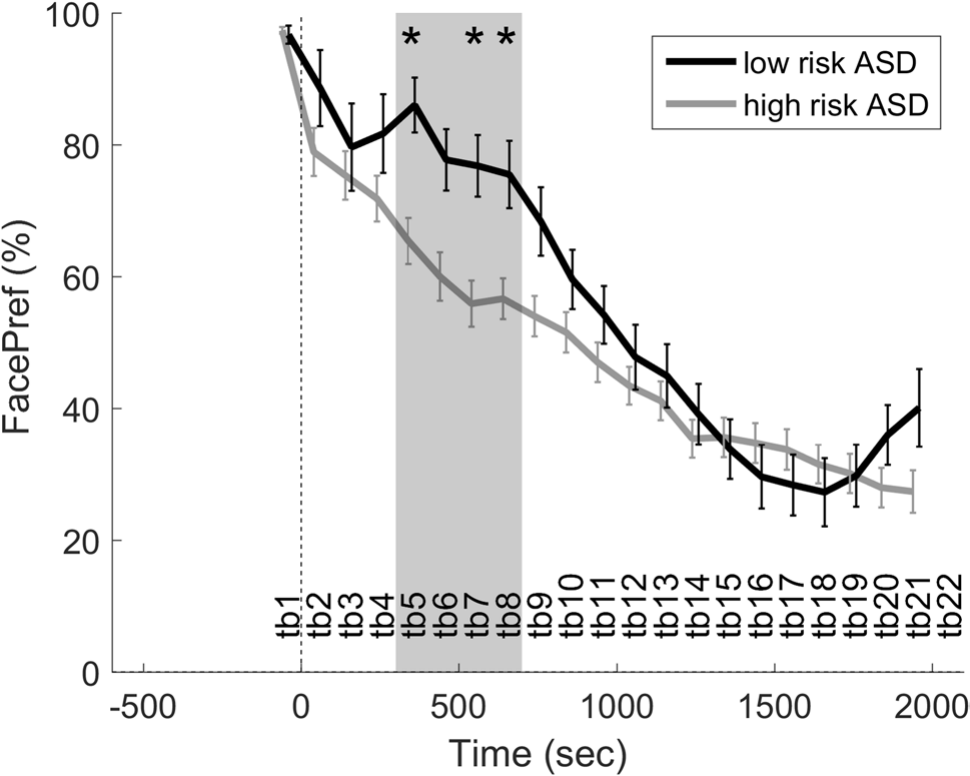
FacePref following direct gaze events (time = 0 s) when the infant was looking at the test leader (baseline >80%). *Error bars* are ±SEM. *Grey* interval includes the time bins with significant differences between groups. The *grey* interval was averaged and tested in a linear regression model. *Asterisks* individual time bins with a significant group difference

Two additional analyses were conducted to investigate the scope of the effect in relation to situations where the direct gaze event was either (primarily) in their central or peripheral visual field. To analyze the former case, a time series analysis was performed using trials where the baseline value was >80%, i.e. the infant was looking at the test leader at the onset of direct gaze (see Fig. 3). Significant differences corrected for multiple testing were found in the interval 300–700 ms after direct gaze. Note that in these analyses, the dependent variable is face preference (FacePref), rather than change in face preference. This interval was averaged within subjects and fit using a linear regression model specified as “FacePref ~ group + model identity”. The results show a significant main effect between groups: F(1, 55) = 6.009, p = .017, R^2^ = .091. Model identify was not significant in this analysis: F(4, 55) = 1.294, p = .28, R^2^ = .078. Descriptive statistics for the dependent variable split by group was: high ASD risk n = 60, M = 59.47, SEM = 3.69, 95% CI 52.03–66.90; low ASD risk n = 18, M = 78.99, SEM = 4.51, 95% CI 69.32–88.67.

**Fig. 4:**
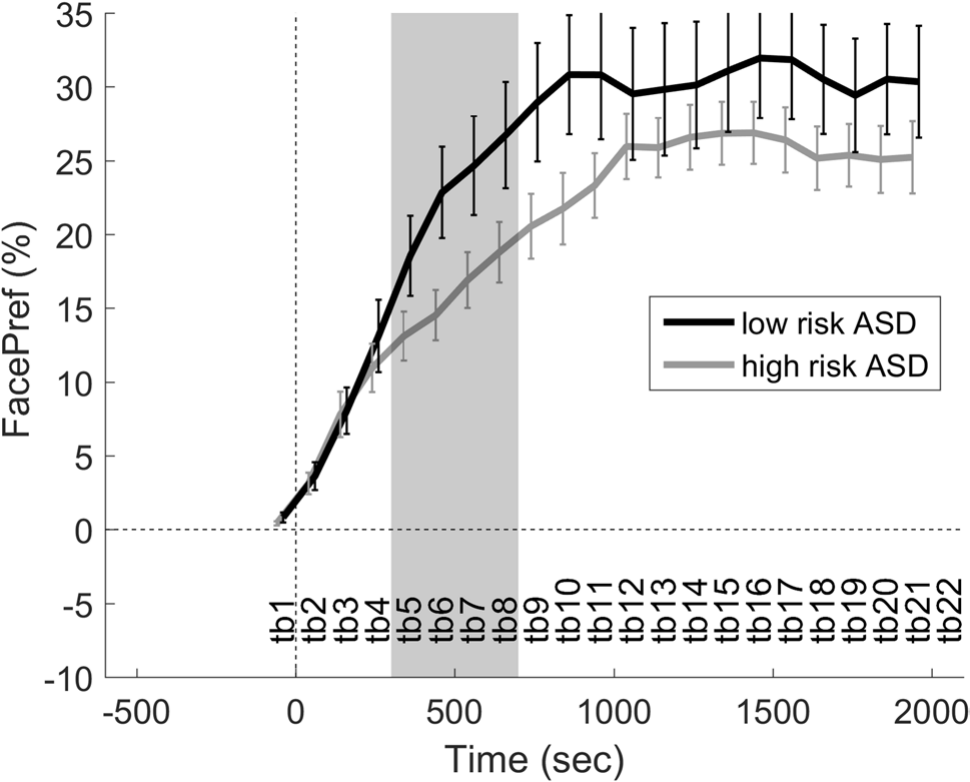
FacePref following direct gaze events (time = 0 s) when the infant was looking at the toy/table (baseline <20%). *Error bars* are ±SEM. The *grey* interval was averaged and tested in a linear regression model

Secondly, a time series analysis was performed using trials where the baseline was <20%, i.e. the infant was looking at the toy/table at the onset of direct gaze (see Fig. 4). There were no significant differences when the *t* tests were corrected for multiple testing, so the same interval as in the previous analysis was used to average FacePref within infants (300–700 ms). Again, a significant main effect was found between groups: F(1, 71) = 4.453, p = .038, R^2^ = .056, but not between test leader identity: F(4, 71) = 1.175, p = .329, R^2^ = .059. Descriptive statistics for the dependent variable split by group was: high risk ASD n = 60, M = 15.83, SEM = 1.72, 95% CI 12.38–19.28; low ASD risk n = 18, M = 23.19, SEM = 3.06, 95% CI 16.73–29.65.

### Looking Time at Longer Timescales

In line with traditional eye tracking paradigms, and to examine overall differences in gaze behavior, we conducted analyses of looking times aggregated over the whole play sessions. An independent *t* test assessing over all face preference (face LT/scene LT) showed no significant difference between the high risk and low risk ASD groups: t(76) = −0.73, p = .465. An independent *t* test of percent looking at the scene (no. of gaze samples in scene AOI/no. of samples in play session), indicated no group differences: t(76) = −1.19, p = .237.

### Additional Control Analyses

Because the stimuli was a naturalistic interaction situation, and because the test leader occasionally uttered words, it is possible that such vocalizations influenced the infants’ gaze differently in the two groups (Shic et al. 2014). To investigate this we compared the percentage of trials with speech in the two groups with a two-tailed *t* test, which did not reveal any significant differences: t(74) = −0.09, p = .930, (high risk ASD M = 55.05, SEM = 2.05; low risk ASD M = 55.40, SEM = 3.75). Two subjects could not be coded due to technical problems with the audio recording. Also, running the time series analysis and regression model with trials without speech gave similar results as the main analysis did but with a shorter time interval (300–700 ms): a main effect was found between groups: F(1, 67) = 6.171, p = .015, R^2^ = .084, but not between test leader identity: F(4, 67) = 1.515, p = .207, R^2^ = .083. Note that in this analysis 4 subjects were excluded because they did not have enough valid trials.

Another possible confounding factor is the eye tracking data quality per se. Because the groups may differ in amount of head and body movement during recording, which could influence data quality over time, we performed a calibration quality assessment between the two blocks. In this assessment a squeaky toy was used to attract the infants’ gaze to the calibration points in the corners of the scene, and the mean distance between the gaze position and calibration point was calculated as a measure of accuracy. A *t* test between the groups showed no significant difference: t(71) = 1.316, p = .192 (high risk ASD M = 35.27px, SD = 10.27; low risk M = 31.64px, SD = 9.76). The standard deviation of the gaze relative the median gaze point was calculated as a measure of precision, and a *t* test between the groups showed no significant difference: t(71) = 1.314, p = .19309 (high risk ASD M = 13.65px, SD = 5.49px; low risk M = 11.77, SD = 4.438). Note that some participants did not provide enough calibration assessment data and was excluded from this analysis. The last data quality control tested the percentage of valid samples in all trials between each group. This t-test was also not significant: t(76) = −0.45, p = .657 (high risk ASD M = 80.43%, SD = 17.55; low risk M = 82.49%, SD = 15.75). Adding these control variables to the main regression did not improve the model, and it is thus unlikely that data quality differences between the groups have affected the main results.

## Discussion

This study shows that immediately after an adult makes eye contact, infants at risk for ASD look less at the adult’s face compared to low risk infants. To our knowledge, these findings are the first to provide evidence for behavioral differences in response to direct gaze in infants at risk for ASD. Moreover, the results reveal the precise temporal nature of the alteration. While infants in both groups increased their looking at the test leader’s face shortly after being looked at (~300 ms after direct gaze; which is a plausible reaction time at this age), the high risk ASD group did so to a lesser extent. Importantly, this effect is not likely to be explained by differences in overall looking at the face versus looking at other objects. The lack of group differences at longer timescales is in line with previous research at this age, e.g. Ozonoff et al. (2010) and Elsabbagh et al. (2012). The time-locked response may thus reveal a difference related to direct gaze processing per se, rather than an overarching difference in face preference. The current findings stress that the overall summation of looking times may sometime miss important information that could otherwise be extracted (Wass et al. 2015).

When analyzing trials where the infants were looking at the test leader at the time of direct gaze (>80% face preference during baseline), the high ASD risk group displayed less looking at the test leader during the analysis interval 300–700 ms. Descriptively, the low risk group had a local maximum (i.e. “positive peak”) during the analysis interval (see Fig. 3), suggesting that direct gaze temporarily reverses the naturally occurring decrease following a period of direct looking at the test leader’s face. In contrast, the high ASD risk group time series showed a relatively linear decrease in face preference that eventually levels out at the end of the trial. If anything, this group seems to have a slight “negative peak” during the same interval, which could indicate gaze avoidance during this period in some of the infants. When analyzing trials where the infants were not looking at the test leader at the time of direct gaze (<20% face preference during baseline, i.e. direct gaze in the periphery), we found a more linear change in both groups, but the increase was more pronounced in the typical group. Thus, the high risk infants’ response is diminished both when the they are looking at the test leader and when the test leader is in the visual periphery at the onset of direct gaze, suggesting that the effects are not only due to differences in orienting responses but also how direct gaze elicits or maintains attention engagement in the different groups (Dawson et al. 2004). Interestingly, the temporal evolution of the gaze response in the current study is reminiscent of the temporal evolution of established neurophysiological measures of attention, in particular the Nc component in infants during direct gaze processing (Hoehl and Striano 2008; Dawson et al. 2002), suggesting that the two measures could be reflecting the same underlying attentional mechanism. The fact that we found similar looking times to the face on longer timescales speaks against reduced social motivation as a likely sole explanation for our results (Chevallier et al. 2012; Dawson et al. 2004).

Previous literature has proposed a number of alternative models for infants’ sensitivity to direct gaze processing, which are reviewed in Senju and Johnson (2009a, b); Senju et al. (2008). In particular the “fast track modulator” model proposes a fast and early developing pathway for detection of faces in the peripheral visual field, and that this pathway facilitates visual attention and oculomotor behavior (2009a, b); Senju et al. (2008). Indeed, even newborns are sensitive to direct gaze and direct their attention to engage in mutual gaze (Farroni et al. 2002). An alteration in this subcortical pathway has been suggested to be linked to ASD (Senju and Johnson 2009a), and our results are broadly consistent with this view (Fig. 4). However, the fast track modulator model has recently been challenged based on other work, which did not find any differences in orienting to peripheral faces in ASD populations (Fischer et al. 2014) including infants with later ASD (Elsabbagh et al. 2013). It is possible that our study detected differences between groups because we used a live situation with a natural interaction instead of monitor presentation, but the differences in results need to be further resolved before any specific model of direct gaze processing can be preferred. Further, with the current study we cannot easily determine whether or not the high risk ASD infants processed the direct gaze events in the same way (reflecting reduced ability), or if they did not respond to these events in the same way as controls despite similar initial detection and processing of these cues (which could reflect reduced motivation to respond).

The present findings may also have practical implications, assuming that response to others’ direct gaze is likely to be important for the development of other social behaviors. Our findings suggest that infants at risk for ASD display alterations in a basic social attention capture function and that the difference is fairly specific (i.e. not paralleled in terms of overall face preference, orienting to name or in poor visual disengagement) and that they generalize across different interlocutor styles (as indicated by the lack of interaction effects between risk status and test leader identity). It is possible that attracting the attention of the infant by other means (e.g. name calling, gestures) during interaction can remedy the potential disadvantage of reduced responding to naturally occurring direct gaze. In practice, this could be done by using other cues of communicative intent such as name calling, gestures, or other means to avoid the potential negative impact on subsequent social abilities. Such negative impact could include that the infants miss important learning opportunities (e.g. verbal naming) or that the caregiver will eventually engage less in mutual gaze and social interaction. Such predictions can be readily tested using longitudinal designs, and should preferably be done so. Clearly the link between response to direct gaze and later outcomes needs to be established before strong conclusions can be drawn, both theoretically and clinically.

As already mentioned, one limitation in the present study is that it is unknown if and how our results relate to later ASD diagnosis. That being said, considering that Elsabbagh et al. (2012) showed that neural responses to direct gaze was altered in infants at risk for autism at a similar age, and that the attenuated response was associated with autism diagnosed at 36 months, it is not unlikely that our study will find a similar association between responding to others’ direct gaze and later emerging ASD. The Elsabbagh et al. (2012) study and the current study together support each other and provide both neurological and behavioral evidence that atypical direct gaze processing may represent a developmental mechanism in ASD that warrants further investigation. A particular strength of the current study is that the results show a difference in behavioral response that may affect the environment (i.e. other people). As noted in the introduction, differential brain activity could not affect other people directly without behavior. However, the exact mechanism behind neurology and behavior is still unknown and both phenomena would need to be recorded simultaneously in order to more definitively link the two.

Another limitation of the present study is the possible reciprocal influence from the response to direct gaze between the test leader and the infant. It is possible that the test leader looked at the infant to respond to the infant’s direct gaze, rather than the other way around. Such occasions may have occurred because we deliberately used a natural interaction situation. However, we believe that following a completely predefined and inflexible monologue and looking pattern would instead undermine the ecological validity of the study. Therefore, when balancing experimental control and ecological validity we opted to emphasize the latter. Further, because of the geometry of the testing space the infant was outside or at the extreme visual periphery of the test leader’s visual field while looking at the object, thereby limiting the infant’s possibilities to influence the test leader. To test whether the test leader responded to the infants, the likelihood of a direct gaze from the test leader within 500 ms of the infants’ onset of direct gaze was calculated. An independent t-test of this measure showed no significant differences between groups: t(76) = 0.29, p = .772. Another way of controlling for this is to test differences in baseline values between groups, because different baselines would suggest that one group looked more at the test leader at the onset of direct gaze than the other group. There was no such difference in trial baseline values between groups: t(76) = −0.05, p = .957, again suggesting that the main statistical analysis was not biased by reciprocal behavior.

In conclusion, infants at risk for ASD are less likely to look at other people’s faces after events of direct gaze compared to typically developing infants in a naturalistic social situation. The control analyses suggest that the underlying mechanism is unrelated to factors such as disengagement times, individual response variability (i.e. including the standard deviation measure of the disengagement control task did not improve the statistical model), face preference, sensitivity to other ostensive cues (calling the infant’s name), and test leader interaction style. The findings are important not only because they provide a fine-grained description of social gaze atypicalities present at 10 months in infants at risk, but also because they describe behavioral differences that could impact subsequent development of socio-cognitive processes via transactional processes. While the results suggest that reduced sensitivity to direct gaze on short timescales early in life is linked to ASD risk, longitudinal research is needed to address the exact relation to later ASD diagnosis.

## Electronic supplementary material

Below is the link to the electronic supplementary material.

Supplementary material 1 (DOCX 442 KB)

Supplementary material 2 (MP4 2901 KB)
