# Supplementary material for: Responding to Other People’s Direct Gaze: Alterations in Gaze Behavior in Infants at Risk for Autism Occur on Very Short Timescales
Source: J Autism Dev Disord. 2017 Sep 4;47(11):3498–509. doi: 10.1007/s10803-017-3253-7 (PMC5633639; doi:10.1007/s10803-017-3253-7)
Supplement: Supplementary file 1 — Supplementary material 1 (DOCX 442 KB) [file 10803_2017_3253_MOESM1_ESM.docx]

# Supplementary information

***Main article:*** Responding to other people’s direct gaze: alterations in gaze behavior in infants at risk for autism occur on very short timescales.

***Authors:*** Pär Nyström, Sven Bölte, Terje Falck-Ytter and the EASE team

As noted in the main article we also investigated whether the main dependent measure is influenced by general attention factors or other measures implicated in ASD. Several studies have shown that individuals with ASD are slower to visually disengage from a stimulus than controls (as shown with the gap-overlap paradigm) (Elsabbagh et al. 2013; Elsabbagh et al. 2009; Landry and Bryson 2004). Also, reaction times may be more variable in autistic individuals than controls (Dinstein et al. 2012; Karalunas et al. 2014) which may affect the main dependent measure in our study.

To assess these factors, we implemented a control task which assessed disengagement time and individual variance for all participants. These measures were used as additional predictors in the linear regression analyses to determine whether the results in the main study remained after controlling for these factors (see main text).

## *Disengagement task*

In the disengagement task, which was performed between the two direct gaze response blocks within the same session, the test leader mimicked a disengagement assessment from the “The Autism Observation Scale for Infants” (AOSI) battery. The infant and the test leader were video recorded (user camera and scene camera respectively, as in the main study), and the infants’ gaze was recorded using the Tobii TX300. During stimuli presentation the test leader lifted and showed an object (a squeaky toy) in the right hand, to the right of the body, until the infant looked at the object. Then the test leader lifted and showed another similar object in the left hand on the left side of the body. The infants usually disengage from the first object and make a saccade to the second object. The objects were then hidden under the table, and the procedure was repeated six times while alternating between starting with the left and right object (thus six trials in total per infant). The dependent measure was the time elapsed from appearance of the second object to the gaze shift from the first object to the second object. The disengagement task took approximately 30 seconds to perform, and a screen shot of the procedure (recorded by the scene camera) can be found in Supplementary Figure 1.


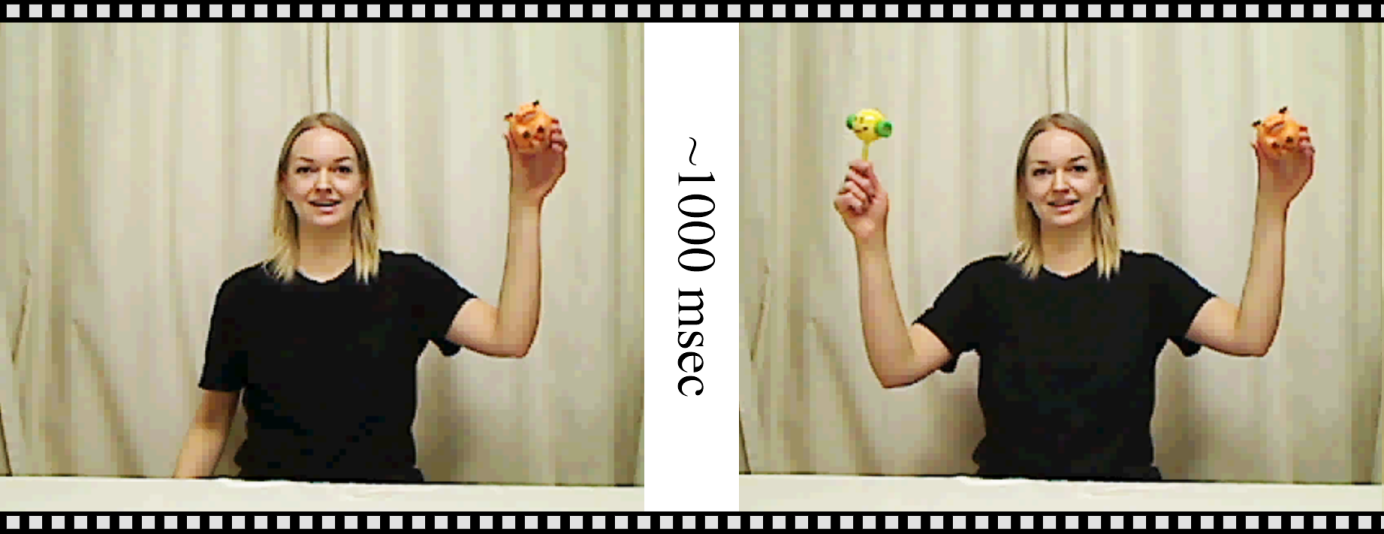


*Supplementary Figure 1. Example of stimuli presentation in the disengagement task.*

The behavior of the test leader and AOI positions were determined by video coding of the scene video using the TobiiStudio software. The recorded eye tracking sessions were exported from the TobiiStudio software as text files and imported into MATLAB (r2015a) and the TimeStudio framework (Nyström et al. 2015). Trials were segmented from the appearance of the first object to 3000 ms after appearance of the second object. Trials with less than 100 ms looking time within the first object AOI before the saccade to the second object AOI were excluded from further analysis. The repetitive nature of the task allowed for predictive gaze shifts after the first trial, but in order to determine the disengagement latency from reactive trials all trials with disengagement latencies <100 ms were also excluded from the analysis. The median latency was calculated within infants and used as our variable for disengagement times. The standard deviation was also calculated within infants and used as the variable for individual variation.

## *Other factors*

Seven more variables unrelated to the disengagement task were used for controlling the response to direct gaze variable in the main task (change in face preference, ΔFacePref).

The first was the baseline value from the main task before baseline correction (e.g. the LT value that was subtracted from all time bins). This measure is important even though overall looking times at the test leader were equal between groups, because the baseline data may contain important between-infant variability.

The second variable was the nominal scoring on the item “Responds to name” from the AOSI which ranges from 0-2, where 0 is scored for “orients (with eye contact) to name being called on both presses, at least one of which must be on the first trial”, 1 is scored for “inconsistent orienting to name (i.e., does not meet criteria ‘0’, but does orient with eye contact on at least one trial” and 2 is scored for “does not orient with eye contact to name on any trial (fails on all four trials with examiner)”. This variable was used to control for sensitivity to other ostensive cues than direct gaze.

The third variable was the Mullen composite score, which was used to control for differences related to overall IQ.

The fourth variable was the number of valid trials, to control for any changes in the dependent variable related to different number of trials between infants.

The fifth variable was the number of trials per second, to control for temporal differences between test leaders’ behavior which could potentially bias the dependent variable.

The sixth variable was the AOSI score from The Autism Observation Scale for Infants , (Bryson et al. 2008), to control for other early ASD traits that could affect the dependent variable.

The seventh variable was test leader identity, as a nominal variable (Female 1-5, and Male 1), to control for behavioral differences between test leaders.

## *References supplementary information*

Bryson, S. E., Zwaigenbaum, L., McDermott, C., Rombough, V., & Brian, J. (2008). The Autism Observation Scale for Infants: scale development and reliability data. *Journal of autism and developmental disorders, 38*(4), 731-738.

Dinstein, I., Heeger, D. J., Lorenzi, L., Minshew, N. J., Malach, R., & Behrmann, M. (2012). Unreliable evoked responses in autism. *Neuron, 75*(6), 981-991.

Elsabbagh, M., Gliga, T., Pickles, A., Hudry, K., Charman, T., Johnson, M. H., et al. (2013). The development of face orienting mechanisms in infants at-risk for autism. *Behavioural brain research, 251*, 147-154.

Elsabbagh, M., Volein, A., Csibra, G., Holmboe, K., Garwood, H., Tucker, L., et al. (2009). Neural correlates of eye gaze processing in the infant broader autism phenotype. *Biological psychiatry, 65*(1), 31-38.

Karalunas, S. L., Geurts, H. M., Konrad, K., Bender, S., & Nigg, J. T. (2014). Annual Research review: Reaction time variability in ADHD and autism spectrum disorders: Measurement and mechanisms of a proposed trans‐diagnostic phenotype. *Journal of Child Psychology and Psychiatry, 55*(6), 685-710.

Landry, R., & Bryson, S. E. (2004). Impaired disengagement of attention in young children with autism. *Journal of Child Psychology and Psychiatry, 45*(6), 1115-1122.

Nyström, P., Falck-Ytter, T., & Gredebäck, G. (2015). The TimeStudio Project: An open source scientific workflow system for the behavioral and brain sciences. *Behavior research methods*, 1-11.
